# Effects of biofertilizers and iron nano‐oxide on maize yield and physiological properties under optimal irrigation and drought stress conditions

**DOI:** 10.1002/fsn3.1884

**Published:** 2020-09-25

**Authors:** Siamak Eliaspour, Raouf Seyed Sharifi, Ali Shirkhani, Salim Farzaneh

**Affiliations:** ^1^ Department of Agronomy and Plant Breeding Faculty of Agriculture and Natural Resources University of Mohaghegh Ardabili Ardabil Iran; ^2^ Crops and Horticulture Research Department Kermanshah Agricultural Resources Research and Education Center (AREEO) Kermanshah Iran

**Keywords:** antioxidant enzymes, drought stress, grain yield, maize, mycorrhiza, pseudomonas

## Abstract

In this research, effects iron nano‐oxide and biofertilizers and chemical was investigated on the yield and some traits of Maize under normal and drought stress conditions in two years (2018 and 2019). The experiment was performed in the form of split–spilt plot in a complete random block design with three replications. The studied irrigation treatment included three levels (normal, 85% and 65% optimum water requirement) in the main plots and iron nano‐oxide at four levels (0, 0.5, 1, and 1.5 g/L) in subplots, and biofertilizers at four levels (noninoculation, inoculation with mycorrhiza, inoculation with pseudomonas and combined inoculation of mycorrhiza and pseudomonas) in sub‐plots. The results showed that grain yield, 1000‐grain weight, and leaf chlorophyll contents decreased by drought stress. Use of pseudomonas and mycorrhiza increased these traits in normal and stress conditions, but iron nano‐oxide had no significant effect on the measured traits. Also, drought stress increased malondialdehyde, ion leakage, catalase, peroxidase, proline, and polyphenol oxidase in both light and severe stress regimes. The amount of antioxidant enzymes increased under drought stress conditions in corn. The results indicated that all the characteristics measured by double inoculation with Pseudomonas and Microoriza had the best performance in conditions of water shortage and the use of these biofertilizers increases yield, 1000‐seed weight, and chlorophyll content of maize. Also, the use of biofertilizers modulates the effect of drought stress and reduces its negative effects.

## Graphical abstract

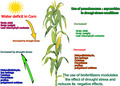

## 1 INTRODUCTION

The growing world's population is constantly in need of food production, and agriculture plays a key role in this production (Eliaspour, Seyed Sharifi, & Shirkhani, 2020; Jahanbakhshi & Salehi, 2019; Jahanbakhshi, Abbaspour‐Gilandeh, & Gundoshmian, 2018; Jahanbakhshi, Yeganeh, & Shahgoli, 2019c; Momeny, Jahanbakhshi, Jafarnezhad, & Zhang, 2020). Maize is considered as one of the most important crops with a variety of uses such as the human and animal food, fodder, and industrial applications. As a food product, it is considered as the main source of the human food in Africa, Latin America, and South Asia. The plant is also the main source of energy in poultry diet in most countries, which is used due to its high energy value, as well as presence of essential pigments and fatty acids (Kaul, Jain, & Olakh, 2003). As a food source, maize provides about 30% of the energy required by more than 4.5 billion people in 94 developing countries. On the other hand, 63% of maize produced in the world is used to feed poultry (Shiferaw, Prasanna, Hellin, & Banziger, 2011). The area under maize cultivation in 2017 in the world was about 200 million hectares with a production of 1 billion 134 million tons. Also, the area under maize cultivation in Iran and in the same year was more than 174 thousand hectares 1,223,000 tons of production. The United States was the largest producer of maize in the world in 2017 with about 33 million and 500 thousand hectares (FAO, 2019). But in 2018, Iran was the seventh largest importer of maize in the world with imports of about $ 1.7 billion of maize and 4.5% of total world imports (FAO, 2019). The factors contributing to the importance of irrigated corn in Iran include its significance in human and animal feeding, its use to produce starch as well as preparing food for poultry farms. In terms of area under cultivation and production, Fars, Khuzestan, and Kermanshah Provinces are the top three respectively. However, in terms of yield per unit area, Kermanshah Province with nine tons per hectare is the first producer. Following wheat, maize is the second most important irrigated crop in Kermanshah Province, with an area under cultivation of 20,000 hectares in 2019.

Iran is the second largest country in the Middle East (after Saudi Arabia) and the 18th largest country in the world with an area of 1,648,195 km^2^. With an estimated population of over 80 million, Iran is the second most populated country in the Middle East (after Egypt) and the 17th most populated country in the world. The average annual precipitation is about 250 mm per year, less than one‐third of the average annual precipitation at the global level. Most of the country receives less than 100 mm of precipitation per year, and 75% of the country's precipitation falls over only 25% of the country's area. Also, 75% of the precipitation is off‐season, that is, falls when not needed by the agricultural sector. Winter is the season with the heaviest precipitation with only few parts of the country (Caspian Sea coast, northwest, and southeast) receiving rainfall in summer. Because of high evapotranspiration and low precipitation, Iran is considered as one of the areas where moisture requirements of maize during the growing season should be met through irrigation water. In many areas, at the most critical stage of growth, that is, flowering and grain filling there is no precipitation. On the other hand, because at this stage the plant is exposed to hot and dry summer climatic conditions and water requirement of other crops is high, so prolonging irrigation periods and/ or postponing 2–3 irrigation rounds are possible at sensitive growth stages. The adverse effects of water shortage on maize growth and yield depend on the time of onset and severity of stress, growth stage, and plant genotype. The results of some studies have shown that water shortage during vegetative growth period compared to water shortage at flowering and grain filling stages has less effect on the final maize yield (AlizadehOqianus, Azeri, & Salimi, 2009). One of the most important factors limiting crop production in arid and semi‐arid regions is water shortage at different stages of growth (Harrison, Tardieu, Dong, Messina, & Hammer, 2014). The negative effects of drought stress have been reported by researchers as the abiotic stress on plant growth (Chaves & Oliveira, 2004) and the most important the abiotic stress affecting crop production in the world (Valliyodan & Nguyen, 2006). The use of biofertilizers is another way to reduce or mitigate the effects of water shortage and increase the microorganisms available in the soil. Mycorrhiza and Azotobacter, as biofertilizers, play an important role in plant feed in mineral humus‐free and poor soils in terms of phosphorus, nitrogen, and other nutrients. These fungi can make nonabsorbable phosphorus available to plants in an absorbable manner and have a significant effect on increasing plant tolerance to drought stresses (Babaei‐Ghaghelestany, Jahanbakhshi, & Taghinezhad, 2020; Eliaspour et al., 2020; Jahanbakhshi & Kheiralipour, 2019). Suitable plant feed under stress conditions can help the plant tolerate various stresses to some extent (Alloway, 2004). Iron is an essential and micronutrient in plants whose deficiency due to reduced chlorophyll content of leaves leads to reduced photosynthesis and rate of carbon dioxide fixation per unit area of the leaf surface (Bisht, Nautiyal, & Sharma, 2002). Nanoparticles foliar application is a useful and effective solution to resolve iron deficiency due to the solubility and greater chance of these particles colliding with the plant (Salehi & Tamaskoni, 2008), high rate of absorption efficiency as well as surface area compared to conventional forms (Monica & Cremonini, 2009).

The application of biological and organic fertilizers in combination with chemical fertilizers is the most important strategy of integrated plant feed for sustainable management of agricultural ecosystems and enhancing their production in sustainable agricultural systems (Ahangarnezhad, Najafi, & Jahanbakhshi, 2019; Delgosha, Mansouri Far, Sadat Asilan, & Asghari, 2015; Eliaspour et al., 2020; Jahanbakhshi, Abbaspour‐Gilandeh, Ghamari, & Heidarbeigi, 2019a; Jahanbakhshi, Rasooli Sharabiani, Heidarbeigi, Kaveh, & Taghinezhad, 2019b). Also, in crop production sustainability plan, soil fertility sustainability is considered as one of the main components. The use of renewable resources and inputs is one of the principles of sustainable agriculture leading to the maximum crop productivity and minimum environmental risks (Kizilkaya, 2008). (Ghorchiani, Akbari, Alikhani, Allahdadi, & Zarei, 2012) showed that under water stress conditions in maize by adding arbuscular mycorrhizal and Pseudomonas fluorescence to the soil, maize yield increased significantly compared to the control that did not have these microorganisms. The coexistence (symbiosis) of microorganisms with the roots of crops boosts the absorption and transfer of moving elements such as mineral nitrogen, especially under water stress conditions. Since the motility of nutrients is low under water stress conditions, arbuscular mycorrhizal can have a significant effect on the growth and development of all plant organs under water stress conditions compared to normal irrigation conditions (Boomsma & Vyn, 2008). Meanwhile, for more than a century, the need for iron to feed plants has been recognized, and iron‐containing fertilizers in crops can be used in different ways. For example, we can mention the methods of soil use, manure pit, and foliar application (Ziaian, 2003).

Nanoparticles and nanocapsules provide efficient means of distributing pesticides and fertilizers in a controlled manner and with a designated location, thus reducing side effects (Nair et al., 2010). Mazaherinia, Astaraei, Fotovat, and Monshi () in their study on the efficacy of conventional iron nano‐oxide and iron oxide on the concentration of iron, zinc, and manganese in wheat stated that iron nano‐oxide, due to higher particle solubility and availability, was mostly absorbed by wheat, which increased the weight of straw, 1000‐grain weight, and the weight of grains in a pot of wheat. This is due to the properties of these materials, including their high specific surface area, solubility, lightness, and smallness, which should be examined in soils with other properties. Fathi Amirkhiz et al. reported that using iron nano‐oxide compared to ordinary iron oxide in wheat, the extent of iron absorption and concentration increased significantly. Moaveni and Kheiri (2011) examined the effect of titanium nano‐oxide fertilizer (TiO2 Nano) on maize growth and yield and stated that this fertilizer has a positive and significant effect on the maize yield. Peykaristan (2015) also stated that using one per thousand nano‐iron fertilizer, the growth and yield of nut maize would increase. Studies have shown that under water shortage stress conditions, use of organic fertilizers and combining nano fertilizers with chemical fertilizers enhances the yield of forage maize. These researchers believed that improving the nutritional status of maize can reduce negative impacts of water shortage stress (Qodrati Avesi, Jalilian, & Siavash Moghadam, 2019). The use of iron nano‐oxide as a new compound of micronutrients is a new way of supplying the plant with the nutrients it needs. Nevertheless, few studies have been conducted on the application of nanomaterials in agriculture, thus necessitating further research in this field (Isivand, Ismaili, & Mohammadi, 2014). The response of plants to water shortage is very complex, which can include a reduction in the relative water content, electrolyte leakage, and production of reactive oxygen species, resulting in membrane damage and inactivation of the enzymatic system. One of the main causes of environmental stress damage to plants is the production of oxygen free radicals. Chloroplasts and mitochondria, two major sites for the presence of electron transfer cycles in plant cells, are always at risk for production of reactive oxygen species (Singh‐Gill & Tuteja, 2019). If these species are activated, oxygen will not be effectively and quickly removed from the plant, which can damage a wide range of lipid cellular macromolecules such as lipids and enzymes (Hui‐Ping et al., 2012). The presence of reactive oxygen species in the cell destroys major cellular macromolecules such as DNA, RNA, and vital enzymes, which are called oxidative damage (Ashraf & Ali, 2008).

Plants with antioxidant enzymatic (superoxide dismutase, catalase, peroxidase, ascorbate peroxidase, and glutathione reductase), nonenzymatic (phenolic compounds, ascorbic acid, glutathione, carotenoids, and alpha‐tocopherols) systems, anions, sugars, and amino acids such as proline, make the membrane structure and various parts of the cell resistant to oxidative stress (Wei, Yang, Wang, & Chen, 2015). Malondialdehyde is a product of peroxidation of unsaturated fatty acids in phospholipids. Thus, to measure the amount of stress applied to plant cells and to determine the involvement of oxygen free radicals as a result of stress, malondialdehyde, which is the result of lipid peroxidation, is measured. A direct correlation has been reported between reducing malondialdehyde concentration and increasing tolerance to salinity stress in wheat (Esfandiari, Shakiba, Mahboob, Alyari, & Shahabivand, 2008). Israr and Sahi (2006) also stated that lipid peroxidation level was used as an index to evaluate the amount of harmful free radicals under stress conditions. Thus, malondialdehyde is used as a reagent to investigate the extent of damage to the membrane under stress conditions. The membrane fats are the primary target of reactive oxygen species, where peroxidation of membrane fatty acids leads to production of malondialdehyde, which is commonly used as a biological index of fat peroxidation and an important index of stress sensitivity in plants (Lata, Jha, Sreenivasulu, & Prasad, 2011). Due to the recent droughts and severe water shortages in Iran on the one hand and the dire need to produce corn grain on the other, it is necessary to study the effect of fertilizers in drought conditions and the possibility of reducing the negative effects of drought stress by biofertilizers. Many researchers have shown that some chemical and biofertilizers can mitigate the negative effects of environmental stresses. This study was conducted to study the effect of mild and severe drought stress on corn as well as the interaction of drought stress and biofertilizers as well as iron nano‐oxide on corn. This is the first time that the effects of biofertilizers and iron nano‐oxide on corn yield in water deficiency conditions on corn in this area have been investigated.

## 2 MATERIALS AND METHODS

The experiment was performed during two years 2018–2019, in the hot area of Kermanshah Province, in the lands of Sarpol‐e‐Zahab training center in Sarpol‐e‐Zahab city (Table 1 Field soil conditions), which is located in the warm climate of the province. The center has a longitude of 45 degrees and 51 min east, latitude of 34 degrees and 30 min north, and altitude of 581 m above sea level (Table 2 Climatic conditions of the region). The experiment was performed in the form of split–split plot design in a complete random block design with three replications. The studied irrigation treatment included three levels of complete irrigation, 85% of the plant's water requirement as mild water stress and 65% of the plant's water requirement as severe water stress in the main plots plus iron nano‐oxide at four levels (0, 0.5, 1, and 1.5 g/L) in subplots and biofertilizers at four levels (noninoculation, inoculation with mycorrhiza, inoculation with pseudomonas, and combined inoculation of mycorrhiza and pseudomonas) in subplots. Glomus imoseaea was used for inoculation for mycorrhiza treatment, which was a mixture of spores, hyphae, and parts isolated from infected roots. The amount of fungi used was 20 g per square mete (g\m^2^) of soil, which was prepared from Turan Biotechnology Company. Also, to inoculate the grains with Pseudomonas Putida sterin 146, an inoculation agent with 107 live and active bacteria per gram was used. Also, a solution of 15% weight‐volume of Arabic gum was used for better adhesion of inoculation to grains. After inoculation, the grains were placed in direct sunlight for two hours and then cultured. These bacteria were obtained from Tehran Soil and Water Institute (AREEO).

The used grain cultivar was KSC201 (Koosha), which is a national early maize hybrid based on a density of 75,000 plants per hectare (area density). Each experimental plot consisted of four six‐meter‐long lines with a row spacing of 75 and a plant spacing of 17.8 cm. Before the five‐leaf stage, optimal irrigation for all plots was carried out in a rainy manner for planting. At the five‐leaf stage, the hydrofix system and water meters were installed, with irrigation applied based on water requirement and the amount of each treatment. From this stage onwards, calculations and measurements were done. The plant's water requirement was calculated based on FAO Penman–Monteith equation and FAO's No. 56 guidelines for 10‐day periods, according to the area's meteorological statistics (Zotarelli, Dukes, Romero, Migliaccio, & Kelly, 2015, Fooladmand, 2011 and Atta et al., 2015), while the common irrigation round In Kermanshah was once every 10 days with the selection of this irrigation round being based on the conditions of the farmers. Software NETWAT was used to calculate water requirement. Based on the input of meteorological data once every 10 days, FAO Penman–Monteith equation, and plant coefficients for different stages, this software calculates plant water requirements. Indeed, this equation has been introduced by FAO experts to calculate water requirement of crops. The amount of water obtained was equal to 100% of water requirement (optimal irrigation) with 65% of this amount considered for low water irrigation treatment or drought stress. Also, according to the irrigation system and the advice of irrigation experts, the system efficiency was calculated 90% with 10% added to the calculated amount of water (Figure 2).

Irrigation water was determined according to the relevant formula (plot area (m^2^) × daily water requirement mm/day × irrigation round).

**Figure 1:**
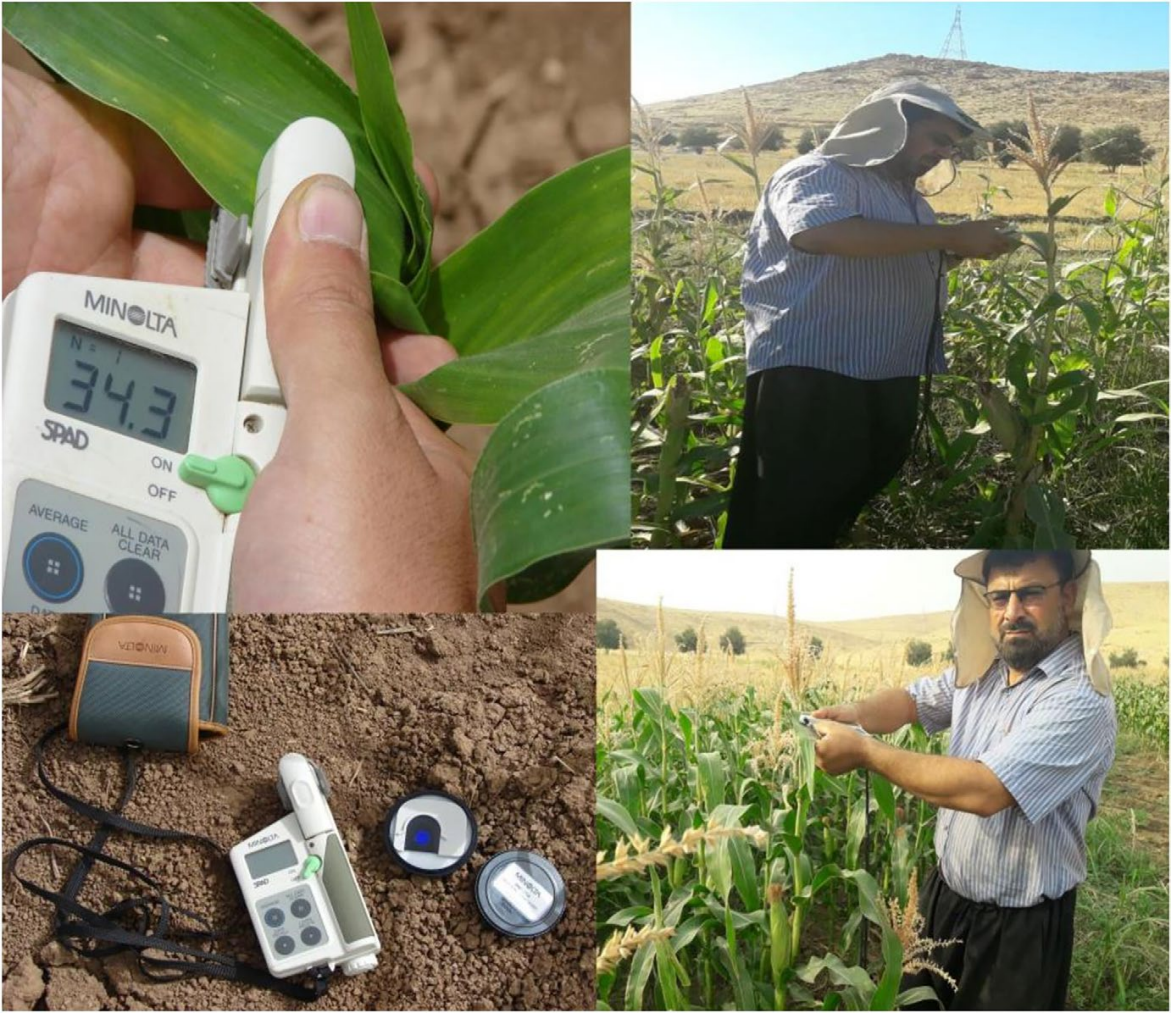
Chlorophyll meter (SPAD‐502, Minolta) readings were taken in all plots

In order to determine the yield, the middle two‐row ears of each experimental plot were harvested in about October 15 and weighed manually at the time of physiological examination, after removing two side lines and two plants from the beginning and end of each plot. The grains were then separated from the ears, the weight of the grain and the ears were determined separately, and finally the grain yield per hectare was calculated in kg. The moisture content of the grains of each plot was determined and recorded separately by a moisture meter. Leaf chlorophyll levels were measured and recorded using SPAD (SPAD‐502, Minolta) at the flowering stage where the maximum leaf area is present (Wilhelm, Ruwe, & Schlemmer, 2000; Schlemmer, Francis, Shanahan, & Schepers, 2005) (Figure 1 in In a farm located in Kermanshah, Sarpol‐e Zahab region).

**Figure 2:**
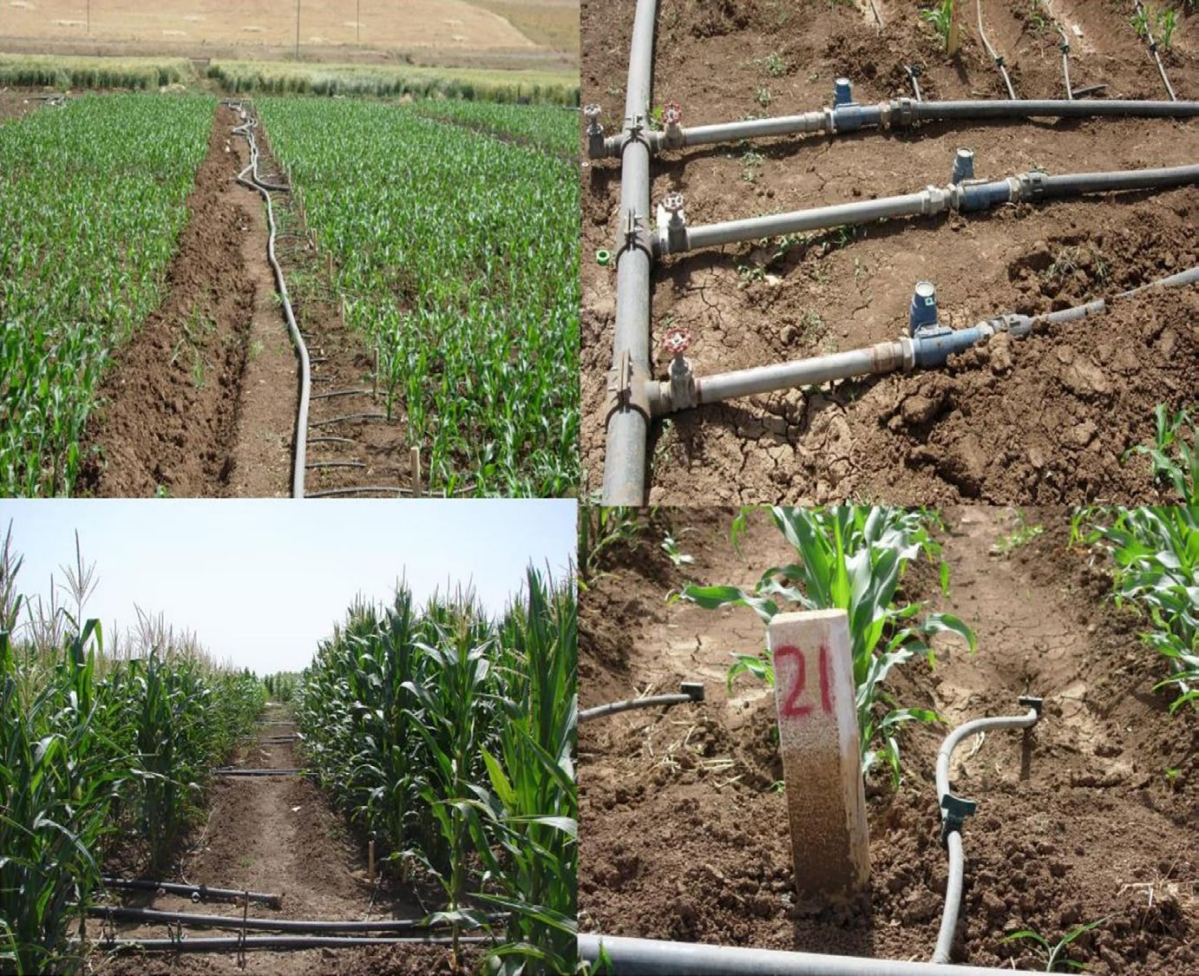
Irrigation system and controlling the amount of water required

**Figure 3:**
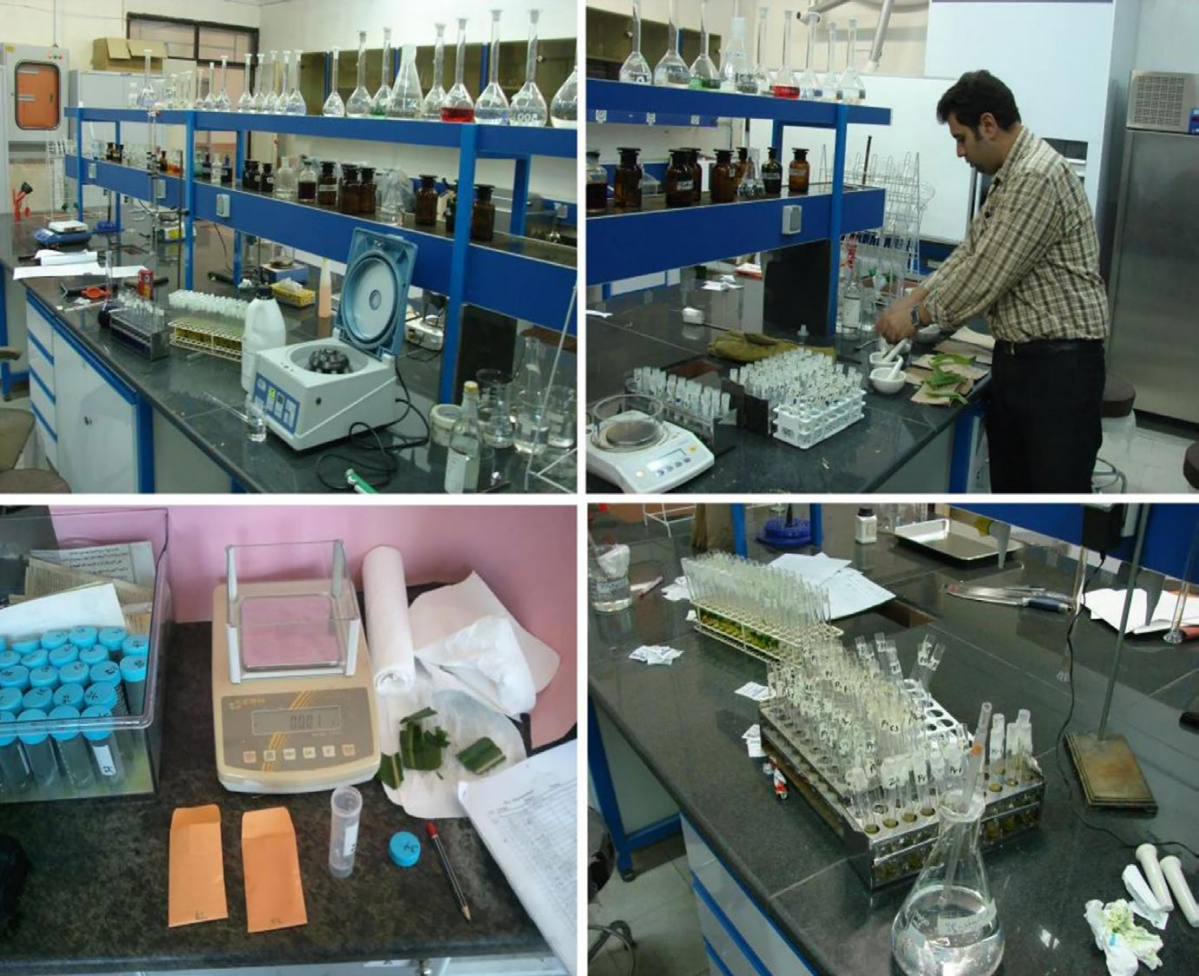
Measurement of enzymes in the laboratory

### 2.1 Malondialdehyde

In order to determine the concentration of malondialdehyde, 1 g of wet tissue was first weighed and homogenized by 2.5 ml of 10% Trichloro acetic acid (TCA) solution. The resulting solution was then placed in a centrifuge for 20 min. Then, an equal volume of 5% trichloro acetic acid and 20% trichloro acetic acid extract was transferred to the test tube and placed in a warm bath at 96 ͦ C for 30 min. The tubes were immersed in ice water for 5 min. The absorption of the resulting solution was then measured by a spectrophotometer at wavelengths of 532 and 600 nm. The amount of malondialdehyde was calculated using Equation (1) (Khazaei et al., 2018):

(1) $$\text{MDA}\left(\frac{\Gamma \text{mol}}{g}\text{Fw}\right)=\left[A532‐\frac{A600}{155}\times 100\right]$$

### 2.2 Catalase

After preparing the protein extract to measure the kinetic activity of catalase (Aebi, 2019), 2.5 ml of phosphate buffer (0.05 and pH = 7) and 0.3 ml of 3% oxygenated water were mixed in ice bath together to which 0.2 ml of enzyme extract was added. Using a spectrophotometer, the absorption change curve was read at a wavelength of 240 nm. The enzyme activity was calculated based on changes in the absorption unit per minute per milligram of protein based on changes in the absorption unit per minute per gram of fresh weight of plant tissue (Mahmoudi, SheikhzadehMosadegh, Zare, & Ismailpour, 2019).

### 2.3 Peroxidase

In order to measure peroxidase, Chance and Maehly (2010) method was used. In this method, the activity of peroxidase was measured based on the formation base of tetragayacol from guaiacol in the presence of hydrogen peroxide and giacol. The reaction mixture consisted of 3 ml of 50 mM potassium phosphate buffer (pH = 7), 50 μg of 20 ml gyacol, 50 μl of 15 mM peroxidase, and 50 μl of cellular extract. After adding the cellular extract, the reduction in absorption was measured at a wavelength of 470 nm for 1 min using a spectrophotometer. The activity of peroxidase was calculated using extinction coefficient (Ɛ = 26.6 mM^−1^cm^−1^) of tetragayacol in ml of enzymatic extract through Equation (2):

(2) $$\text{Enzyme activity}\left(\frac{\text{unit}}{\text{ml}}\right)=\frac{\left[(\Delta A470\text{nm})(3)(df)\right]}{2606\times .05}$$

In this equation, ΔA470 is the amount of adsorption read for each specimen by the spectrophotometer, 3 is the reaction volume, *df* denotes the dilution factor, 26.6 is extinction coefficient of tetragyacol, and 0.05 reflects the volume of enzymatic extract used in ml (Figure 3).

### 2.4 Ion leakage

In order to measure ionic leakage of leaves, the specimens were first rinsed with distilled water and placed in closed tubes. Specifically, 20 ml of distilled water was added to them and placed at 25°C for 24 hr on a rotary shaker. The electrical conductivity of the solution (C_1_) was then measured, and the specimens were placed in an autoclave at 120°C for 20 min, with their electrical conductivity (C_2_) measured again. Ion leakage (EC %) was calculated based on Equation (3) (Lutts, Kinet, & Bouharmont, 1996).

(3) $$\text{El}\left(\%\right)=\left(\frac{C1}{C2}\right)\times 100$$

### 2.5 Proline

Proline was measured based on the method of extraction from plant fresh tissue (Bates, Waldern, & Teare, 1973). This method was first introduced by Bates et al. (1973) and has been revised over time. In this method, 0.5 g of leaves was crushed in a mortar and placed in a tube. Then, 10 ml of 3% sulfuric acid was added to it and the tube was placed in an ice water bath. The tubes were centrifuged where 2 ml of supernatant was separated from it. Also, 2 ml of supernatant was poured into a clean tube, 2 ml of Ninhydrin acid and 2 ml of glycate acid were added to it, and the specimens were placed in a hot water bath at 100°C for 1 hr. The specimens were then placed in cold water to cool. Further, 4 ml of toluene was added to it and stirred. Up to this stage, proline has been extracted and a spectrophotometer has been used to measure it. The spectrophotometer was inserted in specimens using 0 blank solution and read. The absorption rate was calculated using the normal curve of proline value of each sample.

### 2.6 Polyphenol oxidase activity

In order to measure the activity of polyphenol oxidase, Ghanati, Morita, and Yokota (2002) method was used. The reaction mixture consisted of 100 μl of enzyme extract, 500 μg of 5 ml oxygenated water, and 500 μl of 0.2 M methyl catechol per 1900 μl of potassium phosphate buffer. The increase in absorption was calculated at a wavelength of 410 nm and enzyme activity was expressed in mg/ gram of protein/ fresh weight regarding adsorption changes per minute (Tables 1 and 2).

**Table 1:** Physical and chemical properties of soil at the test site

Year of experiment	Soil texture	Absorbable phosphorus (av.P) p.p.m	Absorbable potassium (av.K) p.p.m	Total nitrogen (%)	Organic carbon O.C%	Mnp.pm	Fep.pm	Znp.pm	pH
2018	Silty clay	5.8	480	0.9	1.53	14.5	20.28	3.52	7.35
2019	Silty clay	5.2	500	0.7	1.5	15.8	9.8	3.86	7.50

**Table 2:** Climatic characteristics of the area

Year of experiment	Long‐term precipitation Crop year (mm)	Mean temperature (C)	Max temperature (C)	Min temperature (C)
2018	564.5	22.8	48.6	1
2019	415.5	22.9	48.8	1
Long‐term	406.3	22.5	50.8	−5

## 3 RESULTS AND DISCUSSION

### 3.1 Grain yield

The results revealed that although the use of iron nano‐oxide under optimal and limited irrigation conditions slightly increased grain yield, this growth was not significant (Table 3). However, the interaction between irrigation and biofertilizers on maize grain yield was very significant. By reducing the amount of irrigation water, grain yield was greatly reduced, with the use of Pseudomonas and Mycorrhiza also increasing the grain yield under all irrigation conditions, where the combined use of Pseudomonas and Mycorrhiza had a more positive effect on the grain yield. The highest yield (11.4 tons/ha) belonged to the treatment of complete irrigation and combined use of Pseudomonas and Mycorrhiza, while the lowest yield (4.1 tons/ha) was observed in severe irrigation treatment (65% of plant water requirement) and nonuse of biofertilizers (Table 4). Reduced grain yield under mild water stress conditions was 22.7%, and under severe stress conditions were 56.6% compared to normal irrigation conditions. The results revealed that the use of biofertilizers under conditions of mild and severe water stress can reduce the negative impacts of stress. Also, the positive effect of mycorrhizal was higher than that of Pseudomonas, and a significant difference was observed between these two biofertilizers.

**Table 3:** Analysis of variance of the studied traits under the influence of different irrigation treatments, iron nano‐oxide, and biofertilizers

S.O.V	DF	Grain yield	Spad	Malondialdehyde	Ion leakage	Catalase	Peroxidase	Proline	Polyphenol oxidase
Y	1	0.436 ns	9.39 ns	7.26 ns	0.06 ns	0.001 ns	3.7 ns	0.092 ns	0.002 ns
W	2	883.1**	4,439.2 **	1,600.1**	212.2**	0.726**	10,570.1**	58.08 **	4.88**
W × Y	2	0.9 ns	0.31 ns	0.81 ns	0.152 ns	0.001 ns	4.5 ns	0.073 ns	0.001 ns
Rep (W × Y) E	12	0.209	7.35	0.32	0.027	0.001	9.35	0.018	0.002
Nano	3	0.8 ns	2.62 ns	2.1 ns	0.067 ns	0.001 ns	5.1 ns	0.054 ns	0.006 ns
Y × Nano	3	0.237 ns	0.05 ns	0.564 ns	0.018 ns	0.001 ns	0.99 ns	0.03 ns	0.005 ns
W × Nano	3	0.012 ns	0.32 ns	0.459 ns	0.052 ns	0.002 ns	2.84 ns	0.037 ns	0.003 ns
Y × W × Nano	3	0.012 ns	0.147 ns	0.359 ns	0.01 ns	0.003 ns	1.6 ns	0.032 ns	0.033 ns
Rep × Nano (Y × W) E	36	1.06	7.61	0.652	0.07	0.001	98.9	0.033	0.033
Bio	3	33.06**	41.59**	29.43 **	2.4 **	0.049 **	328.1**	3.62 **	0.5 **
Y × Bio	3	0.361 ns	1.41 ns	0.597 ns	0.029 ns	0.002 ns	5.92 ns	0.019 ns	0.003 ns
Bio × W	6	0.512 **	9.51**	9.73 **	1.47 *	0.035 **	27.77 **	1.16 **	0.292 **
Y × Bio × W×	6	0.043 ns	2.03 ns	1.001 ns	0.069 ns	0.001 ns	2.56 ns	0.01 ns	0.006 ns
Bio × Nano	9	0.02 ns	1.28 ns	0.317 ns	0.016 ns	0.001 ns	2.38 ns	0.043 ns	0.007 ns
Bio × Nano × W	9	0.022 ns	0.187 ns	0.168 ns	0.011 ns	0.001 ns	2.57 ns	0.042 ns	0.006 ns
Y × Bio × Nano × W	18	0.011 ns	0.165 ns	0.217 ns	0.013 ns	0.003 ns	1.44 ns	0.044 ns	0.006 ns
Error	144	0.413	5.741	0.666	0.068	0.001	8.55	0.029	0.002
CV		8.19	6.62	2.76	7.93	9.73	4.48	10.41	11.56

Note

ns, **, and * insignificant and significant differences at probability levels of 1 and 5%, respectively (W: Irrigation, Nano: Iron nano‐oxide, Bio: Biofertilizers, Y: Year, and Rep: Replication).

**Table 4:** Mean of grain yield and Spad of maize in different irrigation treatments and biofertilizers

Irrigation levels (water requirements)	Biofertilizer	Grain yield (ton/ha)		Spad	
100%	Nonapplication	9.93	B	40.88	B
	Pseudomonas	10.05	B	41.66	AB
	Mycorrhiza	11.21	A	42.38	A
	Pseudomonas + Mycorrhiza	11.42	A	42.95	A
85%	Nonapplication	7.45	D	37.02	D
	Pseudomonas	7.75	D	37.6	CD
	Mycorrhiza	7.73	C	38.4	C
	Pseudomonas + Mycorrhiza	8.99	C	38.8	C
65%	Nonapplication	4.14	F	28.16	E
	Pseudomonas	4.22	F	28.21	E
	Mycorrhiza	4.91	E	29.1	E
	Pseudomonas + Mycorrhiza	5.22	E	29.35	E

Zarabi, Allah Dadi, Akbari, Irannejad, and Akbari (2010) also suggested that under conditions of water stress, the negative impacts of stress can be reduced through fertilizer management. Studies by other researchers also show that water stress greatly reduces maize grain yield (Song, Jin, & He, 2019). Seasonal drought is one of the most important factors limiting maize production in the world, with global maize yield diminishing by a mean of 17% each year, though in some areas up to 70% has been reported (Dastbandan Nejad, T, Saki., & Lack, S., 2010). Drought stress at the grain filling stage can reduce the maize yield by 3% per day of water shortage, which indicates sensitivity of maize to irrigation delay. Moisture stress before flowering, at pollination, and grain filling stages can reduce the yield of maize grain by 20 to 50%. Severe water stress reduces leaf area, photosynthesis, chlorophyll content, plant height, stem diameter, and finally grain yield (Bennouna et al., 2004). Drought stress reduces the absorption and transport of nutrients in maize and sorghum. Water stress usually lowers the nutrient uptake by roots and transfer through the roots to the shoots, since the rate of transpiration is limited and impairs the membrane's ability to transmit and penetrate, thereby reducing the root uptake of crops (Ibrahim, Zeid & El‐Semary, 2001). Drought stress changes accessibility of various nutrients in the soil significantly. Thus, plant nutrition management under stress conditions is one of the important issues in production of plant products (Mohammadkhani and Heidari, 2015).

Several studies show the positive effect of biofertilizers on growth and yield of crops such as maize (Ansari, Sarikhani, & Najafi, 2011). According to Zarabi et al. (2010), phosphate solubilizing bacteria (PSB) can increase plant tolerance to water shortage by increasing maize growth and phosphorus uptake. Shirinbayan (2019) showed that under drought stress conditions, the growth and dry weight of maize can be improved using different strains of Azotobacter where negative impacts of stress can be reduced. Ghorchiani et al. (2012) showed that under conditions of water stress in maize by adding arbuscular mycorrhiza and Pseudomonas bacteria to the soil, maize yield increased significantly compared to the control that did not have these microorganisms. The coexistence of microorganisms with the roots of crops increases absorption and transfer of moving elements such as mineral nitrogen, especially under conditions of water stress. As the motility of nutrients is low under water stress conditions, arbuscular mycorrhizae can have a significant effect on the growth and development of all plant organs under water stress conditions compared to normal irrigation conditions (Boomsma & Vyn, 2008).

The study results by Shaharoona, Arshad, Zahir, and Khalid (2005) showed that Pseudomonas bacteria increased the dry weight of maize under greenhouse conditions by 22.5%.

### 3.2 Spad Index

The interaction between water and biofertilizer levels on leaf chlorophyll content was significant, with the results showing that mild and severe water stress significantly reduced the leaf chlorophyll content. On the other hand, the use of biofertilizers increased this trait, but this effect was not significant in severe stress treatment (Tables 3 and 4). The highest leaf chlorophyll content (42.95) was observed in the optimal irrigation treatment and the use of Pseudomonas and Mycorrhiza while the lowest leaf chlorophyll content (28.16) found observed in severe stress treatment. Under conditions of severe stress, biofertilizers did not affect this trait.

Water stress reduces leaf area, photosynthesis, leaf chlorophyll content, and thus, the grain yield (Bismillah Khan, M, Asif., & Aman, M., 2003). Terzi and Kadioglu (2006) also stated that drought stress caused a significant reduction in chlorophyll and carotenoid content as well as chlorophyll stability index. Farnia and Khodabandehloo (2015) also reported that increasing irrigation round from 7 to 21 days, resulting in drought stress in maize, reduced the leaf chlorophyll content. Farnia and Khodabandehloo (2015) reported that mycorrhizal biofertilizer greatly increased the chlorophyll content of maize leaves, and under drought conditions, this fertilizer could enhance the chlorophyll content of maize leaves. Isivand et al. (2014) stated that using iron fertilizer, leaf chlorophyll content in all plants increased. In their study, the level of maize leaf surface index rose using iron nano‐oxide. Ansari et al. (2011) by investigating the effect of nitroxin nitrogen biofertilizers of nitroxin and super nitro pluson maize stated that these fertilizers had no positive effect on leaf chlorophyll index.

The results obtained by Andrade and Ferreiro () on the effect of drought stress on maize showed that with increasing severity of drought stress, the process of chlorophyll stain degradation occurred more rapidly. Gregersen and Holm () stated that drought stress reduced the leaf chlorophyll content, where cultivars with a higher chlorophyll content showed greater tolerance under drought stress conditions. Pessarkli (1999) stated that persistence of photosynthesis and maintenance of leaf chlorophyll under drought stress conditions are among the appropriate physiological indices for tolerance to drought stress.

### 3.3 1000‐grain weight

Grain weight is one of the most important determinants of yield, which is related to the rate and duration of grain filling. In other words, duration of grain filling stage and the rate of transfer of stored materials to the grain as well as efficiency of photosynthesis affect this trait. Reducing the duration of grain filling stage, disrupting photosynthesis, and retransferring photosynthetic materials lower the grain weight (Goodsi, 2004). According to the results, the use of iron nano‐oxide at the rate of 1.5 g per liter increased the 1000‐grain weight from 265.8 to 268.8 g. However, the interaction of iron nano‐oxide with other treatments was not significant. On the other hand, the interaction of different levels of irrigation and biofertilizers on 1000‐grain weight was significant. The results showed that the occurrence of water stress significantly reduced the 1000‐grain weight, but the use of biofertilizers under all water conditions, especially under low water conditions, increased the 1000‐grain weight (Table 4). A significant difference was observed between the effect of Pseudomonas and Mycorrhiza on the 1000‐grain weight under all water conditions, where Mycorrhizal increased the 1000‐grain weight more than Pseudomonas did, where the combined effect of these two fertilizers was greater than the effect of using each alone (Table 5).

**Table 5:** Mean of Grain 1000‐grain weight and Malondialdehyde of maize in different irrigation treatments and biofertilizers

Irrigation levels (water requirements)	Biofertilizer	1000‐grain weight (g)		Malondialdehyde μmol/g Fw	
100%	Nonapplication	285.5	D	15.63	G
	Pseudomonas	292.7	C	15.56	G
	Mycorrhiza	298.5	B	15.46	G
	Pseudomonas + Mycorrhiza	307.3	A	15.54	G
85%	Nonapplication	259.2	H	33.1	D
	Pseudomonas	264.1	G	32.58	E
	Mycorrhiza	271.3	F	32	F
	Pseudomonas + Mycorrhiza	277.7	E	31.79	F
65%	Nonapplication	230.9	L	42	A
	Pseudomonas	233.4	K	41.94	A
	Mycorrhiza	238.5	J	40.47	B
	Pseudomonas + Mycorrhiza	243.7	I	39.29	C

Sajedi and Sajedi (2019) reported that mycorrhiza improved the relationship between water and the host plant by increasing soil hydraulic conductivity as well as transpiration ratio and reducing stomatal resistance by changing the balance of plant hormones; use of this fertilizer improved the maize growth and yield under drought stress. It seems that the use of nanofertilizers can facilitate slow and continuous release of nutrients causing the persistence of leaf area and plant photosynthesis, thereby increasing durability of deployment of growing substances to growing ears and finally resulting in an increase in the 1000‐grain weight. Deficiency of micronutrients significantly reduced the final weight of the grain (Akbari, Mousavi, & SeqaIslami, 2018). Yousefpour & Farajzadeh, 2000) by examining the effect of foliar application of iron and zinc on sweet maize found that foliar application using iron sequestron increased the 1000‐grain weight of maize.

Goodarzi, Kasraei, and Zand (2014) showed that iron micronutrient use enhanced the 1000‐grain weight in maize, and in their opinion, the total content of grain carbohydrates, starch, indole acetic acid, chlorophyll, and protein increased significantly using iron. These factors affect the grain weight. Kamara, Menkir, Badu‐Apraku, and Ibikunle (2003) stated that under drought stress, the maize grain yield dropped by 37% due to 18% reduction in the grain weight and 10% reduction in the number of grains. In studies conducted by other researchers (Mohammadi Behmadi and Armin, 2017), the grain weight and grain filling stage were affected and diminished due to drought stress. El‐Afry, El‐Nady, and Abdelmonteleb (2012) reported that the use of Azotobacterin wheat can act as a protective agent against irrigation water shortage and reduce negative impacts of drought stress.

### 3.4 Malondialdehyde

The results showed that the effect of irrigation levels, biofertilizers, and the interaction between the two were significant on the concentration of malondialdehyde, but iron nano‐oxide had no effect on this enzyme (Table 3). According to the results, with increasing severity of water stress, the concentration of this enzyme increased in the leaf tissue, indicating stress conditions in the plant. On the other hand, the use of biofertilizers, especially the combined use of Mycorrhizal and Pseudomonas, reduced the severity of stress and thus the concentration of malondialdehyde (Table 5). Obviously, under optimal irrigation conditions, biofertilizers had no effect on malondialdehyde. The highest levels of this enzyme were 42 μmol/ g Fw in the treatment of severe stress and nonuse of biofertilizers.

Antioxidant enzymes and malondialdehyde concentrations in plants can be good criteria for evaluating the plant tolerance to drought stress. As such, by measuring the activity of these biomarkers, we can evaluate the tolerance of plants to environmental stresses (Heidari Remi, Moaveni, HosseinpourDarvishi, & Aref Rad, 2016). Studies in sorghum have shown that the concentration of malondialdehyde and dihydroxyguanosine increases during drought stress (Moaveni, 2011). Studies by Asghari and Ebrahimzadeh (2002) on spring wheat showed that under the influence of drought stress at the early stages of growth and development, peroxidase indicated a significant rise. The reason for this increase can be the result of higher sensitivity to optical stress at the early stages of growth and development than drought stress. Gunes et al. (2007) reported an increase in the leaf malondialdehyde concentration under the influence of salinity stress in maize.

### 3.5 Catalase and peroxidase

According to the results of the interaction between irrigation and biofertilizer levels, the activity of catalase and peroxidase was significant, but iron nano‐oxide had no effect on these enzymes (Table 3). As the severity of water stress increased, so did the concentration of these two enzymes while the use of biofertilizers under stress conditions reduced their concentration. Under optimal irrigation conditions, biofertilizers did not affect the activity of catalase and peroxidase (Table 6). Zhu, Song, and Liu (2011) reported an increase in the activity of catalase, peroxidase, and superoxide dismutase in maize inoculated with mycorrhizal fungi. They suggested that mycorrhizal fungi enhanced the antioxidant production, which in turn reduced the number of active oxygen species and protected cells against oxidative stress. The scavengers of reactive oxygen species (ROS) neutralize the toxic effects of reactive oxygen, which may be a result of continuous and simultaneous activity of a number of antioxidant enzymes including catalase, peroxidase, superoxide dismutase, and ascorbate peroxidase.

**Table 6:** Mean of Catalase and peroxidase of maize in different irrigation treatments and biofertilizers

Irrigation levels (water requirements)	Biofertilizer	Catalase od. Min.mg protein		Peroxidase Unit/ml	
100%	Nonapplication	0.142	F	53	G
	Pseudomonas	0.143	F	53.2	G
	Mycorrhiza	0.140	F	53.9	G
	Pseudomonas + mycorrhiza	0.140	F	53.8	G
85%	Nonapplication	0.293	C	67.2	D
	Pseudomonas	0.287	CD	66.5	D
	Mycorrhiza	0.279	CD	64.1	E
	Pseudomonas + Mycorrhiza	0.278	CD	61.9	F
65%	Nonapplication	0.378	A	78.1	A
	Pseudomonas	0.328	B	77.8	AB
	Mycorrhiza	0.270	D	76.2	B
	Pseudomonas + Mycorrhiza	0.216	E	71.7	C

Khalafallah and Abo‐Ghalia (2008) as well as Porcel and Ruiz‐Lozano (2004) reported an increase in the activity of catalase, peroxidase, and superoxide dismutase inoculated with mycorrhizal fungus in their reports. Naseri et al. (2016) by examining the effect of biofertilizers on the physiological properties of wheat under rainfed conditions stated that Pseudomonas putida and G. mosseaein wheat boosted the activity of superoxide dismutase, catalase, and peroxidase. Free‐living bacteria and mycorrhizal fungi have a high and good potential to modulate and regulate the physiological and biochemical responses of the plant to drought stress and hence increase plant survival under harsh and diverse environmental conditions (Mozaffari, Habibi, Asgharzadeh, Bojar, & M., 2016). Omar, Osman, Kasim, and Abd El‐Daim (2009) also reported a reduction in the activity of superoxide dismutase (SOD) in barley plants inoculated with Azospirillum. Erdogan et al. (2016) concluded that plants inoculated with plant growth‐promoting bacteria had a greater antioxidant activity (APX, POD, GR, CAT, and SOD) while the biomarker of malondialdehyde and their hydrogen peroxide was low.

### 3.6 Ion leakage

Obviously, with the occurrence of moisture stress, ion leakage in leaf cells increases. The study results also showed that under mild stress conditions, ion leakage was intensified and with increasing stress severity, the value of this trait was elevated. Note that biofertilizers have a balancing effect and help maintain permeability of the cell membrane under stress (Table 7).

**Table 7:** Mean of Ion leakage, Polyphenol oxidase, and Proline of maize in different irrigation treatments and biofertilizers

Irrigation levels (water requirements)	Biofertilizer	Ion leakage		Polyphenol oxidase mgpr.min		Proline μmole/g	
100%	Nonapplication	16.1	F	0.044	F	0.694	G
	Pseudomonas	16.6	F	0.045	F	0.71	G
	Mycorrhiza	16.4	F	0.044	F	0.694	G
	Pseudomonas + Mycorrhiza	16.5	F	0.044	F	0.633	G
85%	Nonapplication	38.1	D	0.268	D	1.747	D
	Pseudomonas	37.2	D	0.273	CD	1.750	D
	Mycorrhiza	37.3	D	0.156	E	1.583	E
	Pseudomonas + Mycorrhiza	35.1	E	0.150	E	1.355	F
65%	Nonapplication	49.5	A	0.674	A	2.710	A
	Pseudomonas	48.9	A	0.671	A	2.481	B
	Mycorrhiza	42.2	B	0.368	B	1.930	C
	Pseudomonas + Mycorrhiza	40.7	C	0.294	C	1.736	D

Due to the drought stress, permeability of the cell membrane increases and electrolytes inside the cell leak out of the cell. With increasing severity of drought stress, the cell membrane is severely damaged and the cell's ability to control the entry and exit of substances from the membrane is reduced (Mirzakhani & Maleki, 2015). The cell membrane is the primary target of many biological stresses. In general, maintaining the stability of the cell membrane under drought stress conditions is one of the main components of stress tolerance in plants (Dastborhan & Ghassemi‐Golezani, 2015). Electrolyte leakage is also an index of membrane damage and is widely used to study oxidative stress (Silva et al., 2016). Abobatta (2019) also believed that the most important factor in drought tolerance in plants is maintaining the integrity and stability of the cell membrane.

### 3.7 Proline

The results showed that the interaction between different levels of water stress and biofertilizers on leaf proline was significant while iron nano‐oxide fertilizer had no significant effect on this trait (Table 3). Water stress greatly increased the accumulation of proline amino acid in leaves. According to the results, using biofertilizers Pseudomonas and Mycorrhiza, and especially the combined use of Pseudomonas + Mycorrhiza under water stress conditions, the amount of proline in leaves diminished, suggesting the positive and significant effect of these fertilizers on the plant and moderating the negative impacts of water stress. However, under optimal irrigation conditions, biofertilizers had no effect on proline (Table 7). Increasing synthesis of proline amino acid is one of the first responses of the plant to environmental stresses. Increasing proline causes the cell to adapt more to stress conditions and protects the enzymes in cytosol and cellular structures. The accumulation of assimilates in cytosol allows regulating osmotic pressure in the cell and stabilizing enzymes in the presence of ions. The enzymes are also affected and protected by the protein defense mechanism due to their protein structure (Nasrollahzadeh, Shiri, Moharramnejad, Yousefi, & Baghbani, 2016). Chaum, Siringam, Juntawong, and Kirdmanee (2010) examined the effect of different levels of drought stress on the amount of proline accumulation in two drought sensitive and tolerant hybrids of maize. They stated that the rate of proline accumulation in the cell increased with intensifying the drought severity. Anjum et al. (2011) also examined osmotic stress on maize lines and reported that drought stress caused a significant rise in proline across the studied maize lines. Ashraf and Foolad (2007) observed that under conditions of water shortage, the amount of proline accumulation in the root growth zone of maize seedling increased rapidly compared to other amino acids, especially compared to glycine. This suggests that proline may play a role in stimulating root growth under drought stress conditions.

### 3.8 Polyphenol oxidase

The results showed that water stress greatly increased the amount of polyphenol oxidase in maize leaves, but the use of biofertilizers could reduce the negative impact of stress (Tables 3 and 7). Sharghi and KhalilvandBehroozYar (2019) believed that phenolic compounds neutralize free radicals due to their strong antioxidant properties with plants releasing these compounds in response to stresses. Polyphenol oxidase in plant cells plays an important role in oxidation of phenols to quinones and formation of lignin. In experiments under water stress conditions, the amount of this enzyme in maize increased significantly. Zaeem, Niknam, Ebrahimzadeh Maboud, and Sharifi (2016) also reported that drought stress enhanced the activity of polyphenol oxidase significantly in saffron.

## 4 CONCLUSION

Drought stress is one of the major environmental limitation factors for crop productivity. Our results showed that the drought stress caused reduction of yield, 1000‐grain, weight and Spad, but it increased malondialdehyde, ion leakage, catalase, peroxidase, proline, and polyphenol oxidase in both light and severe stress regimes. The amount of antioxidant enzymes increased under drought stress conditions in corn.

The results of the present study revealed that under normal irrigation conditions, as well as mild stress and severe stress, dual inoculation with Pseudomonas and Mycorrhiza had the best effect on the measured characteristics and increased yield, 1000‐grain weight, and corn spade. The use of biological fertilizers also modulated the effect of drought stress and reduced its negative effects. However, iron nano‐oxide fertilizer did not have any significant effect on corn yield under normal and stress conditions. Thus, it is suggested that in case of drought stress, using Pseudomonas and Mycorrhiza biofertilizers, corn grain yield can be enhanced and the negative effects of drought stress can be mitigated.

## CONFLICT OF INTEREST

The authors have declared no conflict of interest.
